# Variation in the seasonality of the respiratory syncytial virus during the COVID-19 pandemic

**DOI:** 10.1007/s15010-022-01794-y

**Published:** 2022-03-22

**Authors:** Lorena Bermúdez Barrezueta, Vanesa Matías Del Pozo, Pablo López-Casillas, Marta Brezmes Raposo, María Gutiérrez Zamorano, María de la Asunción Pino Vázquez

**Affiliations:** 1 División of Paediatric and Neonatal Intensive Care, Department of Paediatrics, Hospital Clínico Universitario of Valladolid, Av. Ramón Y Cajal, 3, 47003 Valladolid, Spain; 2 Division of Neonatology, Department of Paediatrics, Hospital Clínico Universitario of Valladolid, Valladolid, Spain; 3 Department of Paediatrics, Faculty of Medicine, University of Valladolid, Valladolid, Spain

**Keywords:** Bronchiolitis, COVID-19, Pandemic, Respiratory syncytial virus, Epidemiology

## Abstract

**Background:**

The COVID-19 pandemic has caused a variation in the circulation of common respiratory viruses. Our objective was to analyse the epidemiology of respiratory syncytial virus (RSV) bronchiolitis admissions during the COVID-19 pandemic in comparison with previous epidemic seasons.

**Methods:**

We conducted an observational study involving infants with RSV bronchiolitis admitted to a tertiary hospital during two periods: pandemic COVID-19 (15 March 2020–30 September 2021) and pre-pandemic (1 October 2014–14 March 2020). Demographic and clinical characteristics were collected.

**Results:**

A total of 270 patients were admitted for RSV bronchiolitis: 253 in the pre-pandemic period with an average of 42 admissions per season vs 17 in the pandemic. During the pandemic, the RSV outbreak started late in June 2021 with a higher percentage of prematurity and PICU admissions.

**Conclusion:**

A change in RSV seasonality was observed during the COVID-19 pandemic, with an unusual outbreak in summer 2021 of lower magnitude than previous seasons.

## Introduction

Respiratory syncytial virus (RSV) is the main etiologic agent of lower respiratory tract infections in infants and an important cause of hospitalization in this age group. RSV causes annual epidemic outbreaks that in the Northern Hemisphere usually occur between October and March, and is identified in 60–80% of children with acute bronchiolitis [1].

Acute bronchiolitis is clinically defined as the first episode of respiratory distress preceded by catarrhal symptoms with wheezing and/or crackling rales in children under 2 years of age [2]. Bronchiolitis occurs in 20–30% of infants during the first 12 months of life, requiring hospitalization in 2–5% [1].

The COVID-19 pandemic is the greatest health emergency of recent times and has led most countries to adopt restrictive public health measures aimed at controlling its spread, causing a variation in the seasonal circulation of other respiratory viruses [3].

The present study aims to analyse the epidemiology of admissions for acute RSV bronchiolitis during the COVID-19 pandemic in comparison with previous epidemic seasons.

## Methods

We conducted an observational study in a tertiary hospital in north-central Spain, where we compared the frequency of admissions for RSV bronchiolitis during the COVID-19 pandemic (15 March 2020–30 September 2021) in relation to 6 previous epidemic seasons (1 October 2014–14 March 2020) so called the pre-pandemic period. Children under 2 years of age with a diagnosis of acute bronchiolitis according to McConnochie criteria [2] were included, in whom RSV was isolated in respiratory samples by molecular diagnostic tests that detected 17 viruses (Luminex® NxTAG Respiratory Pathogen Panel or FilmArray® Respiratory Panel). In addition, RT–PCR testing for the detection of SARS-CoV-2 was performed during the pandemic.

Data were collected prospectively during the period 2020–2021 and retrospectively by reviewing clinical records in the pre-pandemic period. Demographic and clinical characteristics, comorbid conditions (congenital heart disease, bronchopulmonary dysplasia, cystic fibrosis, Down’s syndrome or neuromuscular disorders) and viral coinfections were analysed.

﻿Data analysis was performed using SPSS v.27. Categorical variables were described as numbers and percentage, and continuous variables as median and interquartile range (IQR). The Mann–Whitney *U* test was used for the statistical analysis of continuous variables and the Fisher exact test or Pearson chi-squared test for categorical variables. *p* values < 0.05 were considered statistically significant.

This study was performed in line with the principles of the Declaration of Helsinki. Approval was granted by the Ethics Committee of Hospital Clínico Universitario de Valladolid, Spain (PI 20-1902).

## Results

During the study period, a total of 360 patients were admitted with bronchiolitis and molecular diagnostic tests were performed in 356 patients. RSV was the predominant etiologic agent in both periods, isolated alone or in association with other viruses in 76.2% of patients in the pre-pandemic stage and 70.8% during the pandemic (*p* = 0.621). The study included 270 patients in whom RSV was identified.

In the pre-pandemic period, there were 253 admissions for RSV bronchiolitis with an average of 42 hospitalizations per season. The beginning and end of each epidemic season ran from November to April or May, with a peak in December or January.

**Fig. 1:**
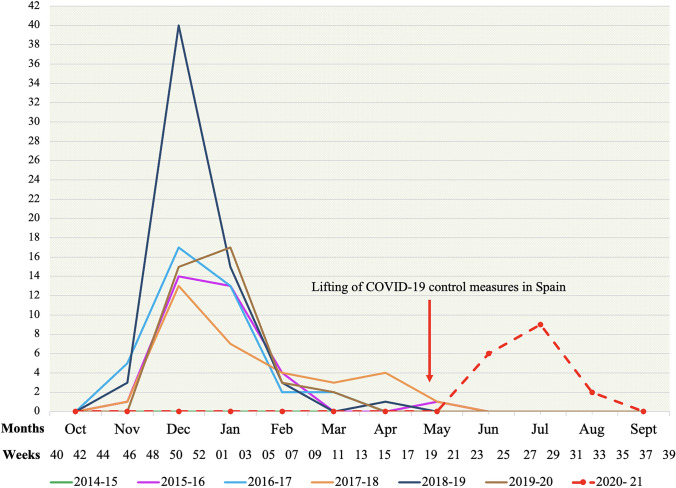
Number of monthly RSV bronchiolitis admissions during each epidemic season and its correspondence with the weeks of epidemiological surveillance. In dashed line, the delayed outbreak of RSV bronchiolitis during the summer 2021 is shown

During the COVID-19 pandemic, there were no patients hospitalized for RSV bronchiolitis during the autumn–winter 2020–2021. The first admission occurred in June 2021, producing an epidemic outbreak that extended through August with a peak in July. This outbreak covering epidemiological surveillance weeks 22 to 33/2021 (peak 27/2021) was smaller in magnitude than in previous winter epidemic seasons, with a total of 17 admissions recorded. Figure 1 shows the number of monthly RSV bronchiolitis admissions during each epidemic season and its correspondence with the weeks of epidemiological surveillance.

We observed that the percentage of viral coinfections associated with RSV decreased from 45.1% in the pre-pandemic period to 29.4% during the pandemic (*p* = 0.21), with RSV and rhino/enterovirus being the most frequent coinfection, with no significant differences between the two periods (23.7% in pre-pandemic vs 17.6% in pandemic; *p* = 0.770). No association of RSV with SARS-CoV-2 was observed.

Concerning demographic and clinical characteristics, we observed that during the pandemic the patients presented lower gestational age, a higher percentage of prematurity, a greater need for admission to the Paediatric Intensive Care Unit (PICU) and use of both invasive and non-invasive mechanical ventilation, compared to pre-pandemic period. Table 1 shows the characteristics of the patients during the two periods studied.

**Table 1:** Characteristics of the patients during the two periods studied (Pre-pandemic and pandemic COVID-19)

	Total	Pre-pandemic	COVID-19 Pandemic	*p *value
	*n* = 270 (%)	*n* = 253 (%)	*n* = 17 (%)	
Age (months)	2.4 [1.2–4.1]	2.4 [1.3–4.1]	1.9 [1–3.6]	0.392
Weight (kg)	5.2 [4–6.3]	5.2 [4.1–6.3]	4.3 [3.4–6.7]	0.187
Sex (male)	139 (51.5)	129 (51)	10 (58.8)	0.531
Birth weight (grams)	3090 [2750–3475]	3090 [2770–3495]	2900 [2430–3402]	0.182
Gestational age (weeks)	39 [37–40]	39 [38–40]	37 [36–39]	0.046
Prematurity	35 (13)	30 (11.9)	5 (29.4)	0.037
Comorbid conditions	17 (6.3)	16 (6.3)	1 (5.9)	1
Siblings	190 (70.4)	177 (70)	13 (76.5)	0.785
Fever	170 (63)	159 (62.8)	11 (64.7)	0.878
^a^Clinical severity score				0.237
Mild	52 (19.3)	50 (19.8)	2 (11.8)	
Moderate	187 (69.3)	176 (69.6)	11 (64.7)	
Severe	31 (11.5)	27 (10.7)	4 (23.5)	
Apnoea	21 (7.8)	19 (7.5)	2 (11.8)	0.63
C-reactive protein (mg/L)	12.9 [2.7–36.1]	12.9 [2.7–32.2]	12.9 [3.8–47.2]	0.643
Viral coinfection	119 (44.1)	114 (45.1)	5 (29.4)	0.208
Coinfections				
RSV-Rhinovirus/enterovirus	63 (23.3)	60 (23.7)	3 (17.6)	0.77
RSV-^b^Coronavirus	29 (10.7)	28 (11.1)	1 (5.9)	1
RSV-Bocavirus	26 (9.5)	26 (10.3)	0	0.386
RSV-Parainfluenza 1-4	18 (6.7)	18 (7.1)	0	0.614
RSV-Adenovirus	9 (3.3)	7 (2.8)	2 (11.8)	3.3
RSV-^c^Influenza	7 (2.6)	7 (2.8)	0	1
RSV-Metapneumovirus	4 (1.5)	4 (1.6)	0	1
RSV-SARS-CoV-2	0	0	0	0
Respiratory support				0.027
None	16 (5.9)	16 (6.3)	0	
LFNC oxygen therapy	107 (39.6)	104 (41.1)	3 (17.6)	
HFNC oxygen therapy	68 (25.2)	64 (25.3)	4 (23.5)	
NIV	76 (28.2)	67 (26.5)	9 (52.9)	
IMV	3 (1.1)	2 (0.8)	1 (5.9)	
PICU admission	90 (33.3)	80 (31.6)	10 (58.8)	0.021
Length of PICU stay	4.3 [3–6.3]	4.3 [2.9–5.8]	5.6 [3–6.9]	0.404
Length of stay (days)	7 [5–10]	7 [5–10]	8 [5–10]	0.269

Categorical variables are expressed as numbers and percentage, and continuous variables as median and interquartile range [IQR]

*LFNC* Low-flow nasal cannula oxygen therapy, *HFNC* High-flow nasal cannula oxygen therapy, *NIV* Non-invasive ventilation, *IMV* Invasive mechanical ventilation, *PICU* Paediatric Intensive Care Unit

^a^Clinical severity score: Bronchiolitis Score of Sant Joan de Deu (BROSJOD Score)

^b^Human Coronavirus type 229E, HKU1, OC43 and NL63

^C^Influenza A, A/H1, A/H1-2009, A/H3 and B

In the pre-pandemic stage, 7 infants (2.8%) were admitted with an indication for immunoprophylaxis with palivizumab according to current recommendations [4]; 6 of them received immunoprophylaxis and none required invasive mechanical ventilation (IMV). During the pandemic, 2 patients (11.8%) had an indication for immunoprophylaxis and only one received a single dose 6 days prior to admission, requiring IMV.

## Discussion

The data presented show a change in the seasonality of RSV during the COVID-19 pandemic. Preventive measures against SARS-CoV-2 (social distancing, use of masks and hand hygiene) have probably contributed to reducing RSV transmission in the winter season 2020–2021, with a late outbreak during summer 2021, which coincided with the relaxation of social restrictions in Spain. This epidemiological phenomenon has also been observed in other countries in different continents. In the northern hemisphere, in France, RSV arrived late in February 2021 and expanded during the spring (weeks 05 to 23/2021), with an outbreak of comparable duration to the previous season, although of lesser magnitude [5]. In the USA, the RSV epidemic started at the end of March 2021 covering the spring–summer months, extending into the autumn in some states [6]. This epidemiological shift was previously contemplated in the southern hemisphere. In Australia and New Zealand, after a 2020 winter season with RSV virtually absent, an unusual reappearance of the virus was observed during the summer, with an even larger outbreak than previous epidemic seasons [7].

In Spain, the Surveillance System for Influenza and other respiratory viruses in week 52/2020 reported only 6 cases of RSV compared to 4578 in the same week of 2019. In week 39/2021, a total of 2001 RSV detections were reported for the entire 2020–2021 season, with the peak incidence observed in week 27/2021 [8]. These data show a variation of the seasonal pattern of RSV in Spain, with a non-existent 2020–2021 winter epidemic season and a rebound of cases in summer 2021, which was 56% lower than the 2019–2020 season. These data are clearly reflected in our study cohort.

The interventions implemented to curb the spread of SARS-CoV-2 have had indirect consequences on the transmission of other respiratory viruses, which explains the lower percentage of coinfections observed during the pandemic, although this finding was not statistically significant in our study.

It is estimated that RSV causes 33.1 million lower respiratory tract infections in young children worldwide each year, generating 3.2 million hospitalizations and 118,200 deaths [1]. Risk factors such as prematurity, congenital heart disease or bronchopulmonary dysplasia are associated with a severe course of the disease. There is no specific treatment for RSV bronchiolitis, and prophylaxis with palivizumab (humanized murine monoclonal antibody specific against the F protein) is the only pharmacological intervention currently approved [4]. This antibody is administered every 4 weeks during autumn–winter in at-risk groups. In our cohort, it was also administered during the summer of 2021, following the recommendations of scientific societies, based on the observation of late outbreaks of RSV in other countries. However, this measure could not be correctly applied in 2 patients who were admitted at the beginning of the epidemic outbreak, one of them suffering from severe disease.

Our study provides data on the characteristics of patients hospitalized for RSV bronchiolitis in an area of Spain. We observed lower gestational age and a higher percentage of PICU admissions during the pandemic, but given the small sample size, it was not possible to establish a causal relationship. Halabi et al. analysed the characteristics of children and adolescents with RSV infection attended in an Emergency Department (USA) during the COVID-19 pandemic, observing a higher proportion of severe disease, need for hospitalization and oxygen therapy compared to previous seasons, without finding an increase in risk factors, such as prematurity or comorbidities [9]. Large multicenter studies analysing the characteristics of children with RSV infection during the pandemic in various health care settings are needed to obtain conclusions with adequate validity.

There is a great expectation about what the next RSV epidemics will be like. Some authors suggest that the mitigated circulation of the virus during the COVID-19 pandemic has led to a decrease in the immune response in children and pregnant mothers, which could lead to the next epidemics of greater magnitude with a higher risk of severe disease. Such a phenomenon has been observed in Australia, which recently reported a high intensity epidemic during the autumn–winter 2021[10].

To our knowledge, this is one of the first studies describing the change in seasonality of RSV during the 2020–2021 season in Spain. Analysing the epidemiological behaviour of RSV during the COVID-19 pandemic and recognizing the beneficial effect that control measures against SARS-CoV-2 have had in reducing virus circulation, is essential to design future outbreak control strategies. There is a need to strengthen epidemiological surveillance systems that alert on circulation trends of RSV and other respiratory viruses, considering the implications this may have on the planning and management of health resources, including the administration of immunoprophylaxis.

## Data Availability

Data is available on request from first author.
